# Combining reward and M1 transcranial direct current stimulation enhances the retention of newly learnt sensorimotor mappings

**DOI:** 10.1016/j.brs.2019.05.015

**Published:** 2019

**Authors:** Danny A. Spampinato, Zabina Satar, John C. Rothwell

**Affiliations:** University College of London, UK

**Keywords:** TMS, TDCS, Reward, Brain stimulation, Motor learning

## Abstract

**Background:**

Reward-based feedback given during motor learning has been shown to improve the retention of the behaviour being acquired. Interestingly, applying transcranial direct current stimulation (tDCS) during learning over the primary motor cortex (M1), an area associated with motor retention, also results in enhanced retention of the newly formed motor memories. However, it remains unknown whether combining these distinct interventions result in an additive benefit of motor retention.

**Methods:**

We investigated whether combining both interventions while participants learned to account for a visuomotor transformation results in enhanced motor retention (total n = 56; each group n = 14). To determine whether these interventions share common physiological mechanisms underpinning learning, we assessed motor cortical excitability and inhibition (i.e. SICI) on a hand muscle before and after all participants learned the visuomotor rotation using their entire arm and hand.

**Results:**

We found that both the *Reward-Stim* (i.e. reward + tDCS) and *Reward-Sham* (i.e. reward-only) groups had increased retention at the beginning of the retention phase, indicating an immediate effect of reward on behaviour. However, each intervention on their own did not enhance retention when compared to sham, but rather, only the combination of both reward and tDCS demonstrated prolonged retention. We also found that only the *Reward-Stim* group had a significant reduction in SICI after exposure to the perturbation.

**Conclusions:**

We show that combining both interventions are additive in providing stronger retention of motor adaptation. These results indicate that the reliability and validity of using tDCS within a clinical context may depend on the type of feedback individuals receive when learning a new motor pattern.

## Highlights

• Concurrently administering reward and M1 tDCS during learning results in enhanced motor retention.
• The combination of these interventions also leads to a reduction in M1 inhibitory mechanisms.
• No benefits of motor retention were found when reward or M1 tDCS were given alone.

## Introduction

Our ability to form motor memories is often taken for granted: it allows us to play the piano, use a smartphone and drive a new car. Yet it is only when movements are impaired after injury or illness that we are reminded of how crucial learned movements are to our daily life. There have been many recent advances in behavioural training methods that optimise performance in elite athletes and restore movement in clinical rehabilitation. Indeed, feedback related to the outcome of the performance (i.e. knowledge of results) is essential for individuals to learn new tasks [[1], [2], [3]]. One commonly used approach is to manipulate feedback about participant performance is to give reward-based feedback during practice [[4], [5], [6], [7], [8], [9], [10]]. Rewarding successful movements has been consistently shown to improve the retention of motor learning in a variety of tasks, and may prove to be a useful strategy to implement in rehabilitation settings. There have also been advances in techniques of non-invasive brain stimulation [[11], [12], [13]], such as transcranial direct current stimulation (tDCS), which improves retention of learning when applied over primary motor cortex (M1) [[14], [15], [16], [17]], an area known to be involved in forming motor memories [[18], [19], [20]]. However, whether one can elicit even greater retention by combining both reward and tDCS remains largely unknown.

One particular type of motor learning in which both reward and tDCS have been shown to have similar effects is motor adaptation. Such tasks involve adapting a movement learned in one context to performance in a novel context, and are typically studied using visuomotor rotation paradigms. This type of learning relies, in-part, on a cerebellar-dependent process [21,22] but also requires M1 for long term retention. tDCS given over M1 during learning enhances the visuomotor retention, so that in the absence of any feedback, the adapted movement returns more slowly to normal. Alternatively, if success during practice is rewarded, retention is also enhanced [[23], [24], [25]]. In this case, the mechanism may involve dopaminergic projections from the ventral tegmental area to primary motor cortex (M1) since these have been shown in animal experiments to mediate the effect of reward in a skill learning task in which rats learned to grasp small pellets from a well with one paw. Thus both reward and tDCS appear able to influence retention of visuomotor learning through an action on M1. The question we address in this paper is whether these interventions can produce additive effects and increase retention more than each method alone.

A possible target for this interaction could be synaptic plasticity. Dopamine release appears to enhance synaptic plasticity in M1, which is critical for long-term M1-dependent motor retention in animals [26,27] and is thought to be a critical process for retention in humans [[28], [29], [30]]. Interestingly, anodal tDCS over M1 is also thought to engage an LTP-like process and enhance synaptic efficacy [31], thus providing a potential shared physiological mechanism that would enhance motor retention. Thus, we tested whether simultaneously administering reward and tDCS during learning would have a greater effect on motor retention than each intervention delivered alone. We also used transcranial magnetic stimulation (TMS) to assess cortical excitability, and to identify any physiological changes that might accompany increased retention. Since both reward and tDCS enhance retention and may have overlapping mechanisms, we hypothesized that their interaction would be additive resulting in stronger retention of the newly learned behaviour.

## Methods

All participants consented to participate in this study and were right-handed, healthy young adults (56 subjects, each group n = 14; age-range 18-35). Participants had no history of neurological diseases nor were there any reports of adverse effects. This study was approved by the research ethics committee of University College London.

## Experimental protocol

**Fig. 1:**
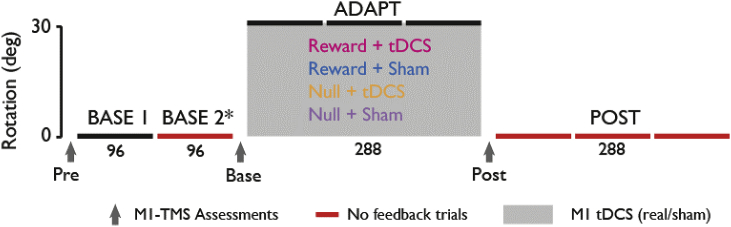
Experimental design. Participants made reaching shooting movements with their right hand toward visual targets presented on a computer screen. The entire experiment consisted of 8 behavioural blocks (horizontal lines, 1 block = 96 trials), separated by three sections: *baseline, adaptation, and no-vision*. The *baseline* trials were administered under veridical conditions (i.e. no visual rotation). During *adaptation* trials (shaded grey region) a 30° clockwise visuomotor perturbation was imposed. Here, half of the participants were given reward feedback in accumulating positive points based on endpoint error (Reward groups), whereas the other half were given end-point visual feedback (Null groups). Moreover, participants from both Reward and Null groups were given either PA-tDCS or sham-tDCS to the left M1 during adaptation (approximately 20 min). Of note, PA-tDCS reflects the positioning of electrodes that were oriented perpendicular to the central sulcus (3.5 cm in each direction). Black horizontal lines represent blocks with online and end-point visual feedback, whereas red horizontal lines represent blocks where no movement feedback was given. Note: there was no movement feedback for all *no-vision* trials. TMS measures were recorded before each of the 3 task parts (black arrows: Pre, P1 and P2). (total n = 56; each group n = 14). (For interpretation of the references to colour in this figure legend, the reader is referred to the Web version of this article.)

All participants underwent a protocol consisting of eight behavioural blocks separated into three distinct parts (baseline, adaptation, no-vision). Each behavioural block contained 96 trials (Fig. 1). Participants were first randomly divided into one of the following four groups: *Reward + Stim, Reward + Sham, Null + Stim, Null + Sham*. The *Reward + Stim* and *Null + Stim* groups both received PA-tDCS over M1 during adaptation, whereas the *Reward + Sham* and *Null + Sham* groups received sham stimulation. Moreover the groups were further separated depending on whether they received reward-feedback during adaptation (*Reward + Stim*, *Reward + Sham*) or not (*Null + Stim, Null + Sham*). TMS measures (M1 excitability, SICI, ICF) were recorded before the start of each behavioural section.

## Behavioural task

Participants used a robotic manipulandum arm developed at the University College of London that is capable of both measuring and controlling the main joints of the arm. Participants were instructed to control the movement of a computer-screen cursor by moving the robotic manipulandum with their right hand in order to make fast, 10-cm shooting movements towards visual targets presented on the screen. Vision of the arm was obstructed from their own view by a mirror that projected a display from a downward facing monitor. This displayed the targets and a cursor representing the position of the right arm in a horizontal plane. Participants were instructed to make rapid “shooting” movements to 3-mm-diameter white targets displayed in one of eight possible positions (25, 70, 115, 160, 205, 250, 295 and 340°), located 10 cm radially from a central starting position. The visual targets appeared in a pseudo-random manner, such that each target position was included in every eight consecutive trials.

The start of a behavioural trial began with participant's placing the cursor at the centre home position (1-cm box). Once participants held this position for 500 ms, a target appeared on the screen at one of eight possible target locations. Participants were instructed to aim to strike through the centre of the target as quickly and accurately as possible. A trial ended as soon as the participant crossed the 10-cm diameter target-space away from the home position. Movement speed feedback was given at the end of each trial to encourage participants to make movements within 200–500 ms: the target turned red or green if movements were either too fast or slow, respectively.

Behavioural blocks were split into three separate categories: *Baseline*, *Adaptation*, and *No Vision*. The *Baseline* section consisted of two blocks (96 trials per block). In the first block, participants were provided with online visual feedback and end-point error feedback relative to the target position (i.e. accuracy). To determine if any movement directional biases existed, the second baseline block was given under veridical conditions (i.e. no visuomotor transformation). The *Adaptation* section involved three behavioural blocks, in which an unexpected 30° clockwise rotation was imposed on the cursor. This visuomotor transformation introduced a performance error which required participants to alter the trajectory of their reaching movements to compensate for the rotation. In these blocks, visual feedback and end-point error feedback was provided for each trial. Participants in the reward groups (*Reward-Stim*, *Reward-Sham*) additionally received an on-screen point scoring system reflecting movement accuracy (4 points: hit the target; 3 points: <10° error; 2 points: <20° error; 1 point: <30° error; 0 points: ≥30° error). Individuals from the reward groups began each block with 0 points and were instructed to improve their score from the previous block point total. The *No Vision* section comprised of three blocks where the rotation was removed, and no visual or endpoint error feedback was provided. These served as the trials used to calculate memory retention by measuring the gradual drift back to baseline performance when visual feedback of performance was removed (“retention” phase). The gradual drift back to baseline performance characterised the degree of memory retention of the learned visuomotor rotation [14,32].

## Neurophysiological assessments

### Transcranial magnetic stimulation (TMS)

We assessed M1 excitability changes by conducting several neurophysiological measures of MEP amplitude, short intracortical inhibition (SICI) and intracortical facilitation (ICF) using TMS. All assessments were performed using a standard figure-of-eight coil (Magstim 200: BiStim^2^, The Magstim Co. Ltd.) on the left hemisphere. We found the ‘hot spot’ for the right first dorsal interosseous (FDI) muscle for each participant. This position was marked on a cap worn by the subjects to ensure identical placement of the coil throughout the experiment. The coil was rotated 45° to the sagittal plane and held tangentially to the skull, over the left M1, inducing a posterior-anterior (PA) current perpendicular to the central sulcus. The resting motor threshold (rMT) was defined as the minimum intensity needed to evoke MEPs of ≥50 μV in 5 out of 10 trials [33]. We then determined the stimulator output intensity needed to evoke MEPs of about 1 mV in peak-to-peak amplitude (s1mV).

SICI and ICF were assessed using paired-pulse TMS with a supra-threshold test stimulus (TS) set to elicit ∼1 mV MEPs and sub-threshold conditioning stimulus (CS) set at 80% of rMT intensity [34]. Standard inter-stimulus intervals were used for SICI (2.5 ms) and ICF (12 ms). After baseline and adaptation behavioural sections were complete, we assessed MEP amplitudes changes by stimulating at the same intensity as used to elicit 1 mV MEP at baseline. This was also repeated for SICI and ICF measures, however, the TS intensity was adjusted to ensure that the MEP amplitudes remained at the same size as before reach movements. MEPs were recorded with electromyography (EMG) using disposable surface electrodes placed over the right FDI muscle and were connected to a Digitimer amplifier. EMG signals were sampled at 5 kHz, band-pass filtered (2 Hz- 2 kHz) and sent to a computer for offline analysis.

### Posterior-anterior (PA) - transcranial direct current stimulation

TDCS was delivered at 1 mA, with a wireless neurostimulator system that triggered stimulation via a Bluetooth receiver (Starstim Neuroelectrics, Barcelona, SP). Contrary to conventional tDCS, where two large 35 cm rectangular electrodes are placed over M1 (anode) and contralateral supraorbital area (cathode), we positioned two small focal 3.14cm2 Ag/AgCl electrodes 3.5 cm posterior (anode) and anterior (cathode) to FDI “hot-spot”. We elected to use PA-tDCS over conventional and 4X1 high definition montage due to a recent study showing this method has effects on the cortex between the primary sites of stimulation [35]. Indeed, effects of PA-tDCS are supported by current-flow models direct which have described current flowing specifically across the central sulcus, thus inducing a PA-tDCS with an electrical field aimed to specifically target M1 [35]. This stimulation yields an average current density of 0.318mA/cm2, a value considered safe and comfortable for stimulating humans [36]. The duration of the stimulation was set to 20 min and was ramped up over 30 s at the onset to ensure subject comfort. Sham stimulation consisted of the initial 30s ramp up, with no subsequent stimulation. These parameters ensured subjects were adequately blinded to the stimulation received [37]. During the experiment two experimenters were present: one delivered the tDCS, whilst the other, oblivious to the type of stimulation continued to run the experiment. This allowed for a double-blinded study, as both the experimenter and participant were unaware of the stimulation being administrated.

## Data analysis

### Behavioural analysis

Task performance was quantified in each trial using endpoint angular error. This was calculated by measuring the angular difference between the centre of the target and the line connecting the starting position to the endpoint hand position [19]. As such, negative values represented clockwise error values whereas positive values indicated counter clockwise (CW) error values. Epochs were created by binning 8 consecutive trials. For each behavioural block, the amount of error (mean) was determined by averaging over consecutive epochs [13]. Each block of 96 trials comprised of 12 epochs, and the entire experiment contained 96 epochs (8 blocks of 12 epochs). For each baseline block the average error was calculated across all trials. In order to assess the different stages of learning and retention we divided the adaptation and no vision blocks into an initial, early and late stage. The *initial* stage was defined as the average endpoint error across the very first eight trials of the respective block. The subsequent 96 trials (i.e. excluding trails 1–8) reflected the *early* stage, and the final 96 trials characterised the *late* stage. Trials in which the endpoint error exceeded three standard deviations of the previous eight trials were considered outliers and discarded.

### Neurophysiological analysis

To asses M1 excitability, the peak-to-peak MEP amplitudes of 12 s1mV single-pulses were averaged before training, and after baseline and adaptation. SICI, and ICF were calculated as the ratio of the mean conditioned over mean unconditioned MEPs. In other words, the ratio of 12 CS + TS (2.5 ms ISI) over 12 TS-alone MEPs was calculated. These analyses were established for each stimulation point (Pre, P1 and P2) and for each individual. Analysis for all TMS assessments was done using Signal version 5.1.

### Statistical analysis

All data analysis was performed using a custom written script in Matlab (Mathworks) and all statistical analysis was performed using SPSS software (SPSS IBM; Version 24). For the behavioural data, mean endpoint error was used as the primary outcome measure and performance was compared between groups using one-way analysis of variance (ANOVA) for the mean of the 2nd Baseline block and both the mean of the Initial (i.e. first block) Adaptation and Retention blocks. Furthermore, to compare how the combination of interventions effects the rates of learning and retention, we used a two-way repeated repeated-measures ANOVA (ANOVARM) with factors between-subjects factor GROUP (*Reward-Stim*, *Reward-Sham*, *Null-Stim*, and *Null-Sham*) and the within-subjects factor TIME (Early, Late). To assess changes in s1mV, SICI and ICF, ANOVARM was used with the between-subjects factor GROUP (*Reward-Stim*, *Reward-Sham*, *Null-Stim*, and *Null-Sham*) and the within-subjects factor TIME (Pre, P1, P2). Mauchly's test of sphericity was used to ensure sphericity. If this assumption was violated, a Greenhouse-Geisser correction was applied. When appropriate, post-hoc comparisons were performed using Bonferroni corrections for multiple comparisons (*p* ≤ 0.05).

## Results

### Reward and tDCS enhance memory retention rates, but not initially

**Fig. 2:**
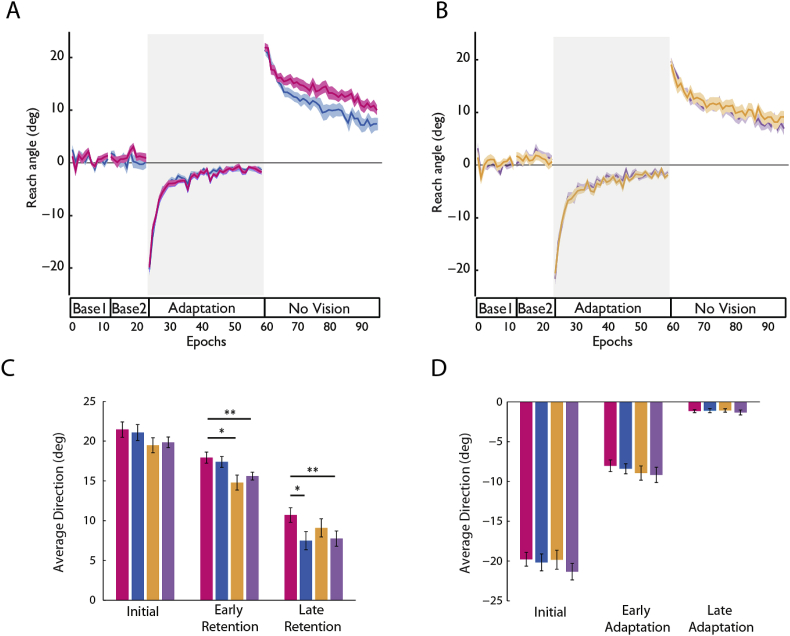
Group behavioural data across the entire experiment (*A and B*) Group behavioural data. Of note, the data are plotted on separate graphs to avoid overlapping lines however analysis considered all four groups. Epochs (x-axis) reflects average error across 8 consecutive trials. Endpoint error (y-axis) during baseline, adaptation and no vision is shown for (*A*) the Reward-Stim (magenta) and Reward-Sham (blue) groups, and (*B*) for the Null-Stim (orange) and Null-Sham groups (violet). Endpoint error was defined as the angular difference between the target position and endpoint hand position. Vertical error bars indicate ± standard error of the mean (SEM) of 8 trial epochs. (*C and D*) Bar graphs indicate mean average endpoint error in degrees (y-axis) during initial, early and late adaptation and retention. (*C*) While no significant differences at initial retention, we found early significant differences for the Reward-Stim group in comparison to Null-Stim and Null Sham groups, and at the end of retention when compared to Reward-Sham and Null-Sham. *P < 0.05 (1-way ANOVA, with Bonferroni's multiple comparison). (*D*) On the other hand, we found no significant difference in either initial, early or late adaptation between groups (1-way ANOVA; *P < 0.05), indicating no enhancing motor adaptation when given reward, tDCS or both in combination. Additionally, this result demonstrates that enhanced retention in the Reward-Stim group is not due to accelerated motor adaptation. (For interpretation of the references to colour in this figure legend, the reader is referred to the Web version of this article.)

We characterised the degree of memory retention as the gradual drift back to baseline performance when the perturbation and visual feedback were removed (No Vision; Fig. 2a and b). To assess *initial retention*, we compared the average endpoint error during first eight trials of no vision. One-way ANOVA did not reveal a significant main effect of initial retention across the groups (*F*_3,55_ = 2.03, *p* = 0.121), suggesting no immediate advantage to administering reward, tDCS or the combination of these interventions towards affecting retention.

However, when we assessed the rate of how well participants held on to the rotation (i.e. early retention = subsequent 96 trials vs. late retention = final 96 trials), ANOVA_RM_ revealed a significant effect for both GROUP (*F*_3,55_ = 5.09, *p* = 0.011) and TIME X GROUP interaction (*F*_3,55_ = 4.14, *p* = 0.019). Specifically for *early retention* phase, *post-hoc* analysis revealed that these effects were driven by differences found between the Reward-Stim and Null-Stim (*p* = 0.011), as well as the difference between Reward-Stim and Null-Sham (*p* = 0.007). This result indicates that the combination of reward and tDCS enhances early retention of a motor memory in comparison to scenarios whereby reward is not received. On the other hand, for *late retention,* Bonferroni post hoc tests revealed a significant difference between the *Reward-Stim* group and *Reward-Sham* (p = 0.041) or *Null-Sham* (p = 0.045) groups (Fig. 2c). In other words, we found a difference between the groups in late retention, where the combination of reward and tDCS produced the largest effect. However, administering either intervention alone does not produce greater retention effects when compared to sham (*Null-Sham)*, suggesting that only the combination of both interventions elicits a prolonged retention and that the effect of reward alone may only influence early retention.

### The effects on retention are not due to differences in baseline or adaptation

To ensure that the differences in retention were not influenced by differences during baseline or adaptation we compared these blocks between the groups. As expected, all groups showed comparable performance during baseline and adaptation (Fig. 2d). There was no significant difference between groups during base1 (*F*_3,55_ = 0.321, *P* = 0.81), base2 (*F*_3,55_ = 0.413, *P* = 0.745), or initial Adaptation (*F*_3,55_ = 0.377, *P* = 0.77),. Similarly, all groups learnt equally as ANOVA_RM_ showed no significant effects for GROUP (*F*_3,55_ = 0.569, *P* = 0.64) nor TIME X GROUP (*F*_3,55_ = 0.952, *P* = 0.42). Therefore, the results of retention cannot be explained by pre-existing baseline differences or in the rate of learning.

## Physiology

### Reduction in M1 inhibitory mechanisms over time, but not M1 excitability or ICF

**Fig. 3:**
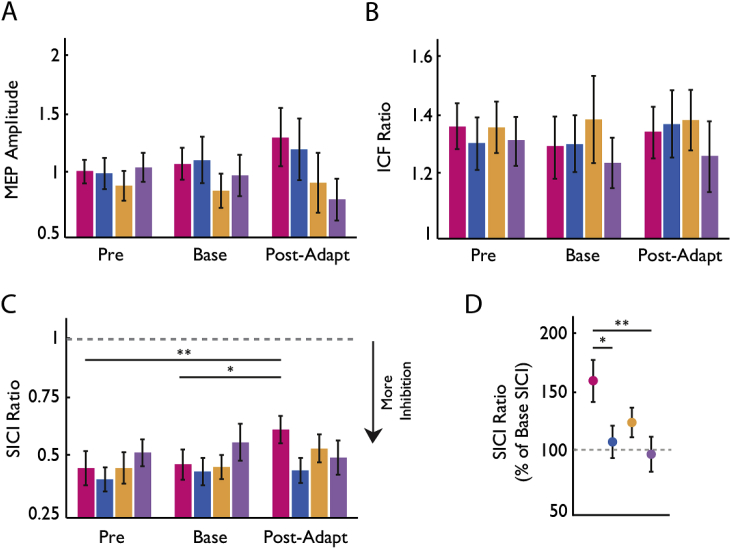
Neurophysiological measures Prior to and Proceeding Motor Learning. The bar graphs show measures of M1 excitability: MEP amplitude in millivolts (mV, A), ICF (B), and SICI (C). The latter 2 are calculated as the ratio of the MEP amplitudes of conditioned over test MEP, times 100, and expressed relative to the test MEP (100%). (*A and B)* Across the groups, we found no significant differences for MEP amplitudes or in ICF following motor adaptation. (*C*) On the other hand, we found that only the Reward-Stim group showed a significant reduction in SICI across stimulation time-points (*p < 0.05). (*D*). Depicts the Mean ± SEM percentage SICI changes versus baseline (base). The SICI ratio significantly changed for the Reward-Stim group in comparison to Reward-Sham and Null-Sham groups (*p < 0.005).

We performed tests of motor cortical excitability consisting of MEP amplitude, SICI, and ICF in the FDI muscle of all subjects (Fig. 3). ANOVA_RM_ revealed significant changes in SICI for both TIME (*F*_2,104_ = 5.497, *p* = 0.005) and TIME x GROUP interaction (*F*_6,104_ = 1.83, *p* = 0.1). Post hoc paired analysis revealed that this result was driven by learning-induced changes in the SICI ratio within the *Reward-Stim* group (*p* = 0.001 and *p* = 0.004). Due to these results, we followed this analysis with a one-way ANOVA in order to determine whether differences in SICI, as a percentage of baseline SICI ratios, varied for each group following learning. Here, one-way ANOVA revealed significant differences between the groups (*F*_3,55_ = 4.383, *p* = 0.008), specifically the Reward-Stim reduced SICI more when compared to *Reward-Sham* (*p* = 0.039) and *Null-Sham* (*p* = 0.008) groups, but not with the *Null-stim* (*p* = 0.286) group. Although we found changes in SICI measures, correlation analysis did not reveal significant relationships between physiological measures and the magnitude of motor retention.

On the other hand, we did not find any significant differences in MEP amplitudes across TIME (*F*_2,104_ = 1.01, *p* = 0.369) or TIME x GROUP interaction (*F*_6,104_ = 1.49, *p* = 0.204) for MEP amplitudes. In addition, we also did not find any significant changes in ICF across TIME (*F*_2,104_ = 0.018, *p* = 0.982), GROUP (*F*_3,104_ = 0.290, *p* = 0.833), or TIME X GROUP interaction (*F*_6,104_ = 0.301, *p* = 0.935). Together these results suggest that the combination of reward and tDCS when learning a new motor pattern modulates inhibitory mechanisms within M1, but not measures of M1 excitability.

## Discussion

Our findings demonstrate that receiving reward-based feedback concurrently with M1 tDCS enhances the formation of new motor memories more than reward or tDCS alone. We also found that SICI was less effective during retention but only when participants had been given both reward and tDCS during learning. These findings suggest that non-invasive brain stimulation can augment the natural behavioural effects of reward. The combined approach might therefore be a useful addition to rehabilitation therapy.

### Reward and tDCS alone are not sufficient to enhance retention

We found that reward-feedback alone did not provide any advantages to either early or late retention when compared to control conditions. This result was surprising given that a recent study had demonstrated that motor adaption is reward-sensitive since retention is enhanced with positive feedback [8]. One key difference is that both TMS and tDCS were administered in this study, which may affect reward-related benefits. For instance, the non-stimulation groups reported here (i.e. Reward-sham and Null-sham) still perceived that they received tDCS since sham-tDCS protocols are administered with a 30s ramp-up and down of current, thus eliciting the sensation of stimulation. This introduces the potential to modify cognitive performance [38], or increase task awareness, which could affect behaviour. In other words, the Null-sham group in this study may have retained more due to the perception of stimulation making participants more attentive during task performance [39]. Alternatively, if the effects of reward are time sensitive, it is possible that the duration of the TMS assessment following learning may interfere with the effect of reward on retention.

We also did not find any advantages to administering tDCS alone in comparison to control conditions throughout the initial stages of retention. While some studies have indicated beneficial effects of M1 atDCS across motor learning tasks [[15], [16], [17],[40], [41], [42]], our results do not yield clear benefits on motor retention. In particular, one study showed that M1 atDCS had no effect on motor adaptation itself but did increase the retention of the new context [14]. However, the critical difference that is important to note is that the present experiments used a more focal tDCS application in comparison to the more classical and conventional approach used previously. Here, we used smaller electrodes that were placed just anterior and posterior to M1, inducing a PA-aligned current [35] in which current flow models have suggested to produce consistent current flow across M1 hand region. Conversely, traditional tDCS involves positioning the cathode over frontal regions, potentially influencing a much larger volume of the brain [43]. Since regions of the frontal lobe, specifically the ventromedial frontal cortex and ventral prefrontal cortex, have been implicated in strategic learning processes [44] that are critical to motor learning [45], it is possible that conventional M1-tDCS engages strategy-influencing brain regions that likely influence motor retention. Given the limited number of studies that have used focal tDCS to augment motor learning, future experiments should explore its relevance and directly compare motor learning in the presence of the two arrangements.

### Reward + tDCS leads to enhanced retention

Recent behavioural work has revealed that the type of feedback given during learning is capable of engaging distinct independent learning mechanisms across motor tasks [4,5,8]. Specifically, reward-based feedback, thought to involve dopaminergic projections from the midbrain to primary motor cortex (M1), has been consistently shown to enhance memory formation of newly acquired motor behaviours [4,6,8], which interestingly is similar to the effects seen on the same tasks when tDCS is applied to M1 [14,16,17,42]. Thus, we predicted that the combination of both interventions may result in greater retention effects. Although combining both interventions did not provide any advantages in immediate retention, it did enhance longer-term memory persistence (i.e. a reduced rate of memory decay) in the absence of visual feedback.

The question that arises is whether reward and tDCS operate on two entirely separate mechanisms which then summate during combined application, or whether they both operate on the same mechanism. Since neither intervention alone had any effect on retention, it seems unlikely that the additional effect of combining them is due to summation of separate mechanisms. Instead, we hypothesized that both reward and tDCS act on LTP-like mechanisms in M1. Reward-related motor learning involves interactions between the ventral tegmental dopamine and M1 [46,47], in which release of dopamine is thought to modulate LTP expression in M1 [48] necessary for motor learning and retention [26,27]. Thus, the augmented retention could be due to reward-related signals strengthening the newly laid down memory trace (i.e. making it more resistant to decay) that are facilitated by enhanced LTP-like effects due to tDCS rather than the summation of two separate mechanisms.

### Reward + tDCS leads to changes in SICI

We did not find any learning-related changes in M1 excitability (i.e. MEP amplitudes and ICF) or SICI (i.e. no changes in *Null-Sham* group). This is probably because the FDI muscle (which is involved in grasping the manipulandum-handle) is not directly involved in the arm movement required during training. Thus we did not expect any changes in excitability since previous studies have shown that these are effector-specific in ballistic-learning tasks [49,50]. Rather, we found that only individuals who were given the combination of reward and tDCS throughout learning modulated SICI. This result suggests that changes in SICI are more sensitive to learning (rather than repeated movement per se) than MEPs. In support of this, one study showed that receiving monetary rewards modulates SICI, but not MEPs when measured after the onset of rewarding visual stimuli [51]. Importantly, all physiological measures were measured in non-involved hand muscles, indicating that the effects of reward + tDCS on inhibitory circuits of M1 are not specific to a muscle involved in the behaviour. Indeed, neither tDCS nor the highly branched dopaminergic projections to M1 are likely to have high spatial specificity. Thus, tDCS may activate representations of multiple muscles, while reward, employing dopamine, may use cortical dopamine projections to GABAergic interneurons [52,53] to modulate their temporal dynamics excitability and excitability [54,55].

Why does the combination of these interventions drive changes in inhibitory mechanisms of M1? One mechanism reward and tDCS likely utilize is LTP-like processes, as both interventions are capable of modulating LTP-like plasticity. GABA has been suggested to have an emerging importance during motor learning [56,57] with its reduction known to be necessary for LTP occurrence [58]. Interestingly, anodal tDCS, thought to involve LTP-like processes, has also been shown to modulate GABA_A_ synapses [59,60]. SICI is recognized to reflect synaptic GABA_A_ receptors within M1 [61], and while the present study did not directly test the effects of reward or tDCS on GABA concentration, it is possible that reduced M1 inhibition resulting in increased LTP-like plasticity would lead to improved memory retention.

## Conclusions

We show that simultaneously receiving reward and tDCS enhances motor retention without affecting acquisition, and moreover this combination of interventions also produced neurophysiological modulation of inhibitory networks within M1. These results have implications for the use of reward-feedback and stimulation as retention enhancing tools. Future studies on patients could investigate the long-term effects of the interventions and how they may translate to settings whereby more complex behaviours are being learnt. For instance, enhancing retention within and between motor-therapy sessions could shorten the extensive contact hours needed for motor improvements.

## Conflicts of interest disclosure

The authors declare no competing financial interests.
